# Impact of Reduced Dietary Crude Protein in the Starter Phase on Immune Development and Response of Broilers Throughout the Growth Period

**DOI:** 10.3389/fvets.2020.00436

**Published:** 2020-08-07

**Authors:** Mohammad Kamely, Wanwei He, Jeremy Wakaruk, Rose Whelan, Victor Naranjo, Daniel R. Barreda

**Affiliations:** ^1^Department of Agricultural, Food and Nutritional Science, University of Alberta, Edmonton, AB, Canada; ^2^Department of Biological Sciences, University of Alberta, Edmonton, AB, Canada; ^3^Evonik Nutrition & Care GmbH, Hanau, Germany

**Keywords:** broiler, cytokines, dietary crude protein, inflammation, leukocyte, innate immunity

## Abstract

Crude protein (CP) levels in commercial broiler (*Gallus gallus*) diets, optimized for maximum yield production vs. feed cost, have only begun to be assessed for impact on immune function. In order to study immune effects of dietary CP levels, different starter phase (day 1–14) diets were fed to 230 Ross 708 male broiler chicks randomly assigned at 1 day of age into two treatment groups. Group 1: Standard diet (STD) contained 3,000 kcal AMEn/kg energy and 23.78% CP; and Group 2: Reduced crude protein diet (RCP) contained 3,000 kcal AMEn/kg energy and 21.23% CP. From day 15–35 a common standard grower/finisher diet (3,150 kcal AMEn/kg energy and 22.18% CP) was allocated to both groups. Zymosan, a glycan derived from yeast cell walls that binds to TLR 2 and Dectin-1, was used for intra-abdominal challenge. Results demonstrated that a reduced crude protein starter diet (21.23 vs. 23.78% CP) between age 1–14, while maintaining the same levels of metabolizable energy and essential amino acids, did not affect broilers growth performance or lymphoid organ weights (*P* > 0.05). Interestingly, basal leukocyte levels in the RCP group significantly (*P* < 0.01) increased in the blood compartment at d35 in the unchallenged birds. Significant enhancements to leukocyte infiltration into the abdominal cavity were also detected post-immune challenge with zymosan (day 14 and day 35; *P* < 0.01). Post-challenge levels of TNF-α, IL-1β, and CXCL8 gene expression cells collected from the abdominal cavity were not affected by the diets (*P* > 0.05). Moreover, dietary treatments did not influence percentage of ROS producing cells in the abdominal cavity (*P* > 0.05). To our best knowledge, this is the first study that reports the impacts of reduced crude protein diet on the innate immune response of poultry to an acute inflammation model in the abdominal cavity. Overall, our results highlight that reduced crude protein diets can be used without negatively impacting broiler performance and may enhance the capacity of broilers to recruit leukocytes upon infection.

## Introduction

Broilers have been exposed to intense genetic selection for enhanced performance traits, primarily for fast growth and efficiency of feed utilization, and thus may have sustained detrimental effects on the immune system (1, 2). Already, differences have been detected in the adaptive immune system (3, 4). For example, the 1957 broiler strain has higher levels of anti-SRBC antibodies (total Ig and IgM) than the 1991 strain when challenged with SRBC at 2 weeks of age (5). Innate immune responses, crucial to limit pathogen diffusion following entry into a host (6), are less known regarding changes in functionality due to genetic selection. In one example, acute-phase protein response remained unchanged among the broilers from 1970, 1990, and modern broilers (7). In contrast, heterophil function showed high variability among genetically distinct lines in respect to phagocytosis, heterophil extracellular traps, and cytokine expression (8, 9).

Another important factor which modulates immune function, while interacting with genetic make-up, is the composition of diet. Dietary protein sources are often a high expense component of poultry diets. As animals require specific amino acids, formulations requiring specific amino acids often lead to crude protein (CP) levels that are overly costly and result in excess nitrogen being excreted as an environmental pollutant (10). The industry has therefore taken strides to formulate diets on a digestible amino acid basis, rather than formulating on crude protein levels, which are much less precise (11). The resulting reduction in crude protein is made possible through the supplementation of the diet with crystalline amino acid sources to supply sufficient limiting essential amino acids (11–13). While extensive research has been conducted to determine the standard ileal digestible amino acid requirements for all feeding phases of broiler chickens in healthy conditions (14), the requirements for optimal immune development, or during a challenge, remain largely unknown (15). Additionally, since nutrient inclusion rates in commercial diets are usually no greater than needed to maximize efficiency of production, it is generally not known if this is sufficient to maintain a robust immune system.

Observation of immune responses following *in vivo* intra-peritoneal/intra-abdominal injection of zymosan has already added to our understanding of immune status in comparative animal studies including fish, pigs, mice, and chickens (16–18). In this model, zymosan serves as a fungal immune stimulator, and under self-resolving conditions allows examination of induction and control phases of acute inflammation. The abdominal cavity of birds provides a suitable environment to study the contributions of migrating leukocytes to the induction and regulation of immune antimicrobial responses. The phenotype and numbers of infiltrating leukocytes can be examined following an *in vivo* immune challenge, and infiltrating leukocytes can be readily harvested for *ex vivo* examinations. For this study, this model was used to evaluate broiler growth and immune function, comparing broilers raised on reduced crude protein starter diet vs. a standard commercial crude protein level diet for the starter phase. Growth and immune function were monitored for 35 days.

## Materials and Methods

### Animals and Experimental Design

A total of 230 male Ross 708 broiler chicks (Sofina Foods, Lilydale Division, Edmonton Hatchery) were randomly assigned at 1 day of age into two dietary treatment groups: standard crude protein (STD) and reduced crude protein (RCP). In order to maximize data quality and minimize stress-associated impact, parallel pens were used for immunological and performance measurements. Eight pen replicates were used in total, with six pens of 15 birds (three pens per treatment) for growth performance measurements (total of 90 birds) and two pens of 70 birds (one pen per treatment) for immunological parameters (total of 140 birds). Dietary treatment groups were set up in an alternating pen pattern within the barn. All birds were housed in the Poultry Research Facility of the Department of Agricultural, Food and Nutritional Sciences at the University of Alberta. All animals were maintained according to the guidelines specified by the Canadian Council on Animal Care, and protocols were approved by the University of Alberta Animal Care and Use Committee.

Diets (crumble starter, pelleted grower) and water were provided *ad libitum* throughout the experiment. From d 1–14 two different starter diet with different CP levels were allocated, with amino acid ratios balanced based on Evonik recommendations (AMINOChick®). The RCP diet contained 3,000 kcal AMEn /kg and 21.23% CP, while the STD contained 3,000 kcal AMEn /kg and 23.78% CP. From d 15–35 the same standard grower/finisher diet (3,150 kcal AMEn /kg and 22.18% CP) was allocated to all groups. The feed formulation and nutritional composition of the starter (1–14 days of age) and grower (15–35 days of age) diets are shown in Table 1.

**Table 1:** Composition of basal diets in different phases of the experiment.

**Ingredients (%)**	**Starter diet (days 1–14)**		**Grower diet (days 15–35)**
	**Standard CP^a^**	**Reduced CP**	**Standard CP**
Corn	51.31	59.30	61.13
Soybean meal, 48% CP	40.45	32.91	29.90
Soybean oil	3.90	2.67	4.73
Dicalcium phosphate, 19%	2.25	2.28	1.97
Limestome	0.66	0.70	0.69
Vitamin Premix Vit+Min	0.50	0.50	0.50
Salt	0.31	0.31	0.31
MetAMINO (DL-Methionine)	0.29	0.35	0.26
Biolys (54.6% L-Lysine)	0.13	0.46	0.23
Sodium bicarbonate	0.09	0.09	0.10
Choline chloride, 60%	0.06	0.10	0.10
ThreAMINO (L-Threonine)	0.04	0.14	0.07
ValAMINO (L-Valine)	-	0.12	0.03
L-Isoleucine	-	0.07	-
**FORMULATED NUTRIENT COMPOSITION,% AS IS (UNLESS STATED)**			
AMEn^b^ (kcal/kg)	3,000	3,000	3,150
Crude protein	23.78	21.23	22.18
Calcium	0.96	0.96	0.87
Available Phosphorous	0.50	0.50	0.44
SID^c^ Lysine	1.24	1.24	1.04
SID Methionine	0.60	0.63	0.52
SID Methionine + Cysteine	0.90	0.90	0.78
SID Threonine	0.79	0.79	0.68
SID Tryptophan	0.26	0.22	0.21
SID Arginine	1.49	1.27	1.18
SID Isoleucine	0.91	0.85	0.73
SID Valine	0.98	0.98	0.83
**ANALYZED NUTRIENT COMPOSITION (% AS FED)**			
Crude Protein	24.60	21.97	20.31
Lysine	1.45	1.37	1.22
Methionine + Cysteine	0.98	0.95	0.85
Threonine	0.98	0.92	0.83
Arginine	1.63	1.41	1.30
Isoleucine	1.07	0.96	0.85
Leucine	2.05	1.85	1.73
Valine	1.18	1.09	0.97
Histidine	0.63	0.56	0.52
Phenylalanine	1.22	1.08	1.00
Glycine	1.00	0.89	0.83
Serine	1.19	1.06	0.98

a *CP, crude protein*.

b *AMEn, apparent metabolizable energy*.

c *SID, standard ileal digestible*.

### Body Weight Gain, Feed Intake, and Feed Conversion Ratio

Body weight was measured weekly for each pen. Feed intake was determined as the difference between the amount of feed offered and the amount unconsumed in starter and grower phase. The daily feed intake (DFI) was calculated by dividing each pen's consumed feed on starter and grower phase by actual total number of birds. The feed conversion ratio (FCR: g feed/g body weight gain) was calculated by dividing daily feed intake by daily body weight gain.

### Lymphoid Organ Weights

The femur bone, thymus, bursa of Fabricius and spleen weight were measured at 7, 14, 28, and 35 d of age from five birds per group. These organs and the corresponding chicks were weighed, and organs relative weight was expressed as a percentage of body weight (BW).

### Intra-Abdominal Injections

Induction of an intra-abdominal acute inflammation was derived from a custom protocol previously validated in our laboratory (18). Briefly, zymosan A from *Saccharomyces cerevisiae* (2.5 mg, Sigma-Aldrich) was resuspended in 500 ml of PBS^−/−^ (no calcium/no magnesium) and administered through injection into the abdominal cavity to induce inflammation response. Birds that did not receive an injection at 0 h were used as the control group. This injection dose allowed the establishment of self-resolving conditions, where early induction and late resolution phases of acute inflammation could be observed.

### Isolation of Peripheral Blood, Bone Marrow, and Intra-Abdominal Leukocytes

Whole peripheral blood samples were taken by wing vein venipuncture. In order to determine the short- and long-term immune impacts of diets, total leukocyte numbers, heterophil, monocyte/macrophage, and lymphocyte populations were measured at 0, 4, and 12 h after zymosan intra-abdominal injections on d 14 and 35 in the bone marrow and abdominal cavity. Bone marrow leukocytes were obtained after femur collection, where bones were flushed with 20 ml of sterile RPMI-1640 medium. Red blood cells were removed from blood and bone marrow samples through treatment with a red blood cell lysis buffer (ACK Gibco, USA) (1:10 ratio). Intra-abdominal leukocytes were recovered by injecting 10 and 20 ml of RPMI-1640 medium on d 14 and 35, respectively. Cells were harvested and maintained at 4°C. Chickens that did not receive an injection were used as a negative control (0 h birds). Blood total leukocytes were counted in a hemocytometer chamber using Natt and Herrick's solution to obtain a 1:200 blood dilution (19).

### Microscopy and Cell Staining

Leukocytes (1 × 10^5^ cells) from peripheral blood, bone marrow, and intra-abdominal were spun onto glass slides at 55 g for 6 min at room temperature using a cytocentrifuge (Thermo Scientific ™ Cytospin ™ 4) and stained using the Hema3 staining set (Fisher Scientific) according to the manufacturer's specifications. Images were generated using a DM1000 microscope (Leica, Wetzler, Germany) and a brightfield 100× objective (1,000× total magnification).

### CellROX Oxidation and Antibody Staining

ROS production was determined based on the CellROX oxidation procedure (Molecular Probes). Briefly, 1 × 10^6^ intra-abdominal leukocytes were incubated with 5 μM of CellROX reagent, followed by 30 min incubation at 41°C. Leukocytes were washed twice with PBS^−/−^ and fixed in 1% formaldehyde. Data were acquired on an ImageStream MKII multispectral imaging flow cytometer (Amnis Corporation, EMD Millipore, Seattle, WA, USA) and analyzed using INSPIRE software (Amnis Corporation, EMD Millipore; Seattle, WA, USA).

### Gene Expression

Gene expression was performed as previously described (17, 20). Briefly, abdominal lavage cells were collected with RPMI 1640 and centrifuged and kept within 1 mL Trizol reagent (Invitrogen Life Technologies) in liquid nitrogen. Total RNA was extracted and reverse-transcribed into cDNA using SMARTScribe Reverse Transcriptase (Clontech) according to manufacturer's protocols. Quantitative PCR was run on ABI 7500 system (Applied Biosystems, Carlsbad, CA, USA). RNA concentration and purity were determined by using a NanoDrop spectrophotometer and 2100 Bioanalyzer. The data were normalized to chicken from time 0 h. The ribosomal RNA 28S gene was used as an internal and normalization. Each sample was performed in triplicate. Relative expression levels for target genes were calculated according to 2^−ΔΔCt^ method. The primer sequences for each gene are shown in Table 2.

**Table 2:** Primers used for QPCR analysis of chicken mRNAs.

**RNA target**	**Sense**	**Sequence**	**Accession number**
28s	Fw	GGCGAAGCCAGAGGAAACT	FM165415
	Rv	GACGACCGATTTGCACGTC	
CLCX8	Fw	GGCTTGCTAGGGGAAATGA	AJ009800
	Rv	AGCTGACTCTGACTAGGAAACTGT	
TNF-α	Fw	GTTGACTTGGCTGTCGTGTG	AY765397.1
	Rv	TCAGAGCATCAACGCAAAAG	
IL-1β	Fw	AGGTCAACATCGCCACCTAC	NM_204524.1
	Rv	ACGAGATGGAAACCAGCAAC	

### Statistical Analysis

Evaluation of significance was based on two-way analysis of variance (ANOVA) analyses with time point and treatment as main effects, using Sidak's multiple comparison tests in Prism 8 software (GraphPad Software, La Jolla, CA, USA). Additional Tukey's *post hoc* tests were also performed for analyzing the differences within the treatments and time after intra-abdominal challenge. Bursa of Fabricius, thymus, femur, spleen, body weight, feed intake, and feed conversion ratio data were analyzed by *t*-test. Statistics with *P* < 0.05 were considered significant.

## Results and Discussion

### Performance and Lymphoid Organ Weights

Reduced dietary crude protein in the starter phase of broilers did not significantly affect body weight gain, feed intake, or feed conversion ratio in either the starter phase or beyond until 35 days of age (*P* > 0.05; Table 3). Broilers fed an RCP diet tended to have lower body weight gain between d 1 and d 28 but compensated between d 28 and d 35 (Table 3). This is consistent with previous research which has shown that reduced protein starter phase diets do not have a negative impact on performance, and also, lower body weight will be compensated after the fourth week (19, 21, 22). This is also consistent with prior reports which point to specific amino acid supplementation rather than CP as most critical to broiler performance. Maintenance of optimal amino acid ratios for essential amino acids and sufficient glycine equivalent levels appear most important (13, 14, 21, 23–25). Based on this, feeding RCP diets formulated to meet digestible amino acid requirements could be an efficient way to reduce nitrogen excretion to the environment and decrease feed cost without impacting growth performance.

**Table 3:** Broiler daily body weight gain, daily feed intake and feed conversion ratio (FCR) during starter (d 1–14) and grower (d 15–35) phase of broiler growth (Mean ± SEM).

**Items**	**STD**	**RCP**	***P*-value**
**DAILY FEED INTAKE (g)**			
Day 1–14	37.61 ± 1.2	33.86 ± 0.8	0.06
Day 15–35	136.1 ± 4.3	137.3 ± 7.5	0.89
**DAILY BODY WEIGHT GAIN (g)**			
Day 1–14	29.4 ± 1.3	27.0 ± 0.2	0.13
Day 15–35	94.7 ± 0.5	96.0 ± 3.5	0.73
**FCR (g/g)**			
Day 1–14	1.28 ± 0.04	1.25 ± 0.02	0.61
Day 15–35	1.44 ± 0.04	1.43 ± 0.03	0.88

*(three pen replicates, 15 birds each). RCP, Reduced Crude Protein; STD, Standard diet*.

**Figure 1:**
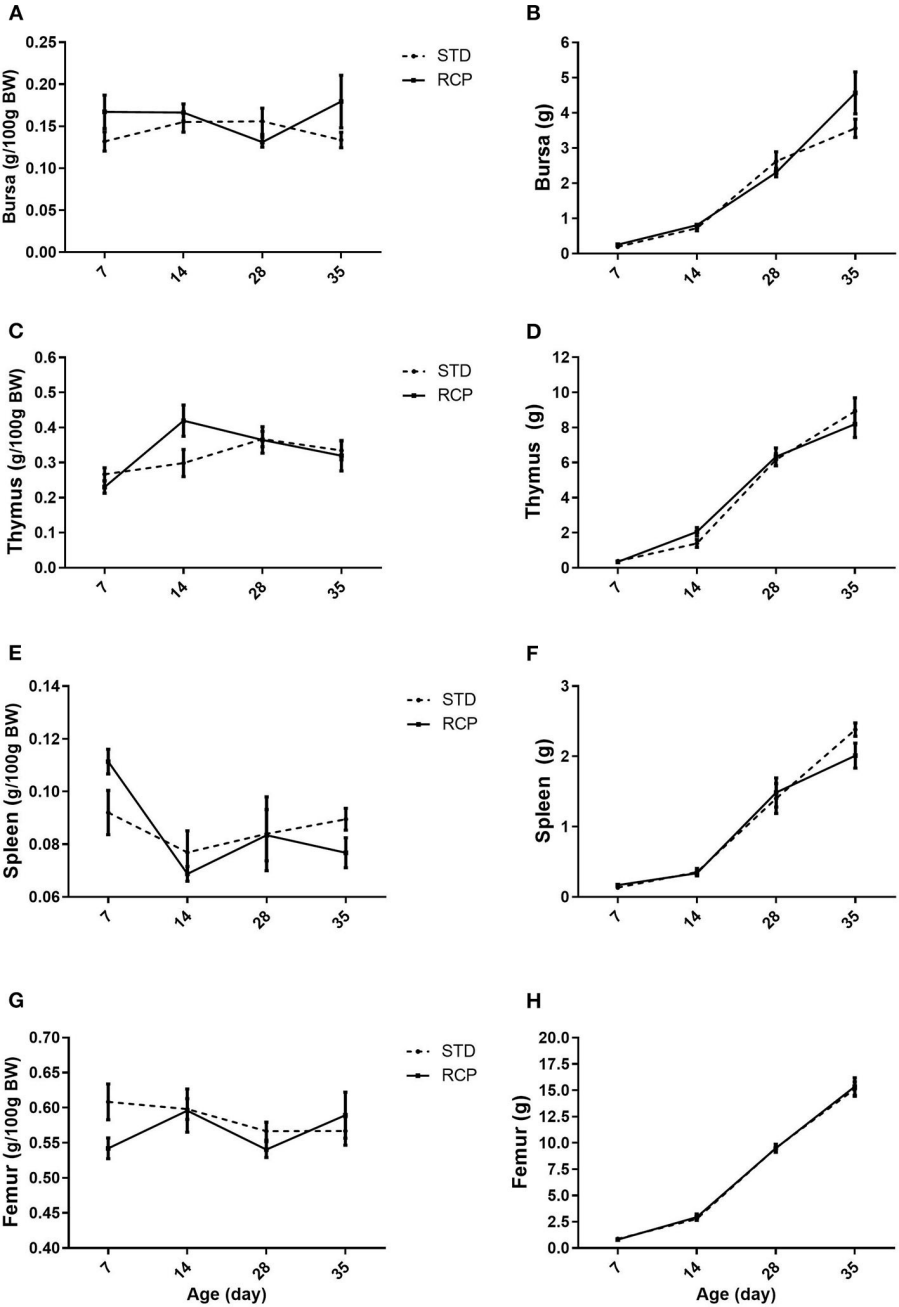
Impact of reduced crude protein diet on chicken lymphoid organs relative weights (g/100 g BW). Bursa **(A)**, thymus **(C)**; spleen **(E)**, femur **(G)**, and lymphoid organs absolute weight (g), Bursa **(B)**, thymus **(D)**; spleen **(F)**, femur **(H)**, on day 7, 14, 28, and 35 of trial (*n* = 5 per group). RCP, Reduced Crude Protein; STD, Standard diet.

The bone marrow and spleen constitute immune relevant organs that, among others, harbor stores of mature naïve leukocyte populations that can be rapidly deployed upon infection (5, 6). The primary lymphoid organs in chickens (bursa and thymus) provide the developmental environment for B and T cells, respectively (26), and deficiency of amino acids or excess of dietary protein has been shown to change immune response in poultry (27). However, RCP diet supplementation did not affect lymphoid organ development (Figure 1). No significant differences were noted across bursa, thymus, spleen, or femur, suggesting no deficiency in those nutritional components that are required for proper development of lymphoid organs. This is consistent with previous research that showed that moderate decrease in CP level (range between 18 and 23%) had no effect on bursa, thymus, and spleen weight (19, 28, 29).

### Leukocyte Recruitment Into the Abdominal Challenge Site and ROS Production After Zymosan Challenge

**Figure 2:**
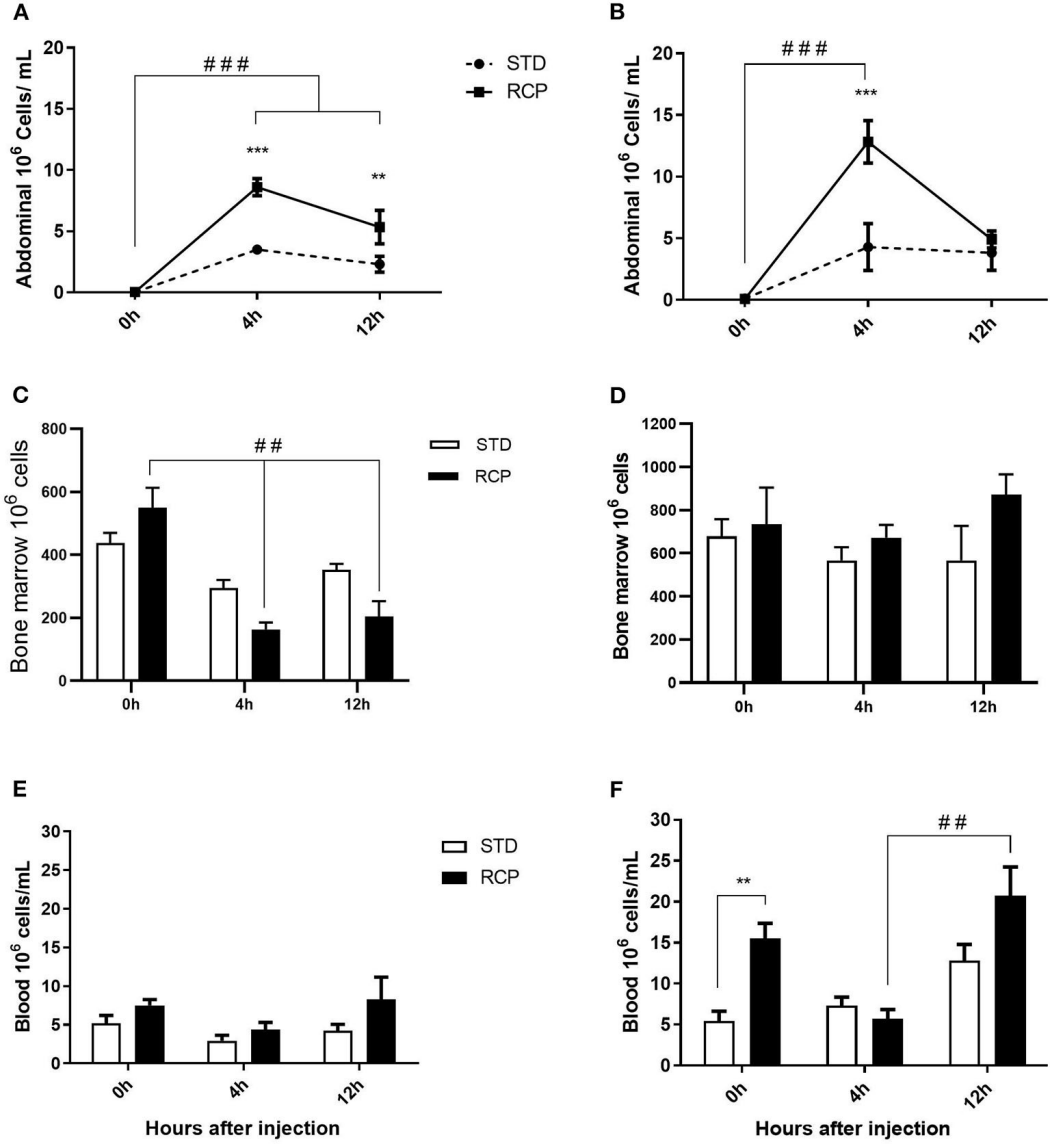
Impact of reduced crude protein diet on leukocyte migration following an *in vivo* zymosan challenge. Recruitment into the chicken abdominal cavity when *in vivo* challenge was performed on d 14 **(A)** or d 35 **(B)**. Cells were harvested by abdominal lavage at 0 h (without injection) and at 4 and 12 h post intra-abdominal administration of zymosan (2.5 mg), (*n* = 5 for time 0 and 12, and *n* = 6 for time 4 h). Corresponding changes to the number of leukocytes in the bone marrow on day 14 **(C)** and 35 **(D)**, and the peripheral blood on day 14 **(E)** and 35 **(F)**. **P < 0.01 and ***P < 0.001 between dietary treatments and ##*P* < 0.01 and ###*P* < 0.001 among different time points. RCP, Reduced Crude Protein; STD, Standard diet.

**Figure 3:**
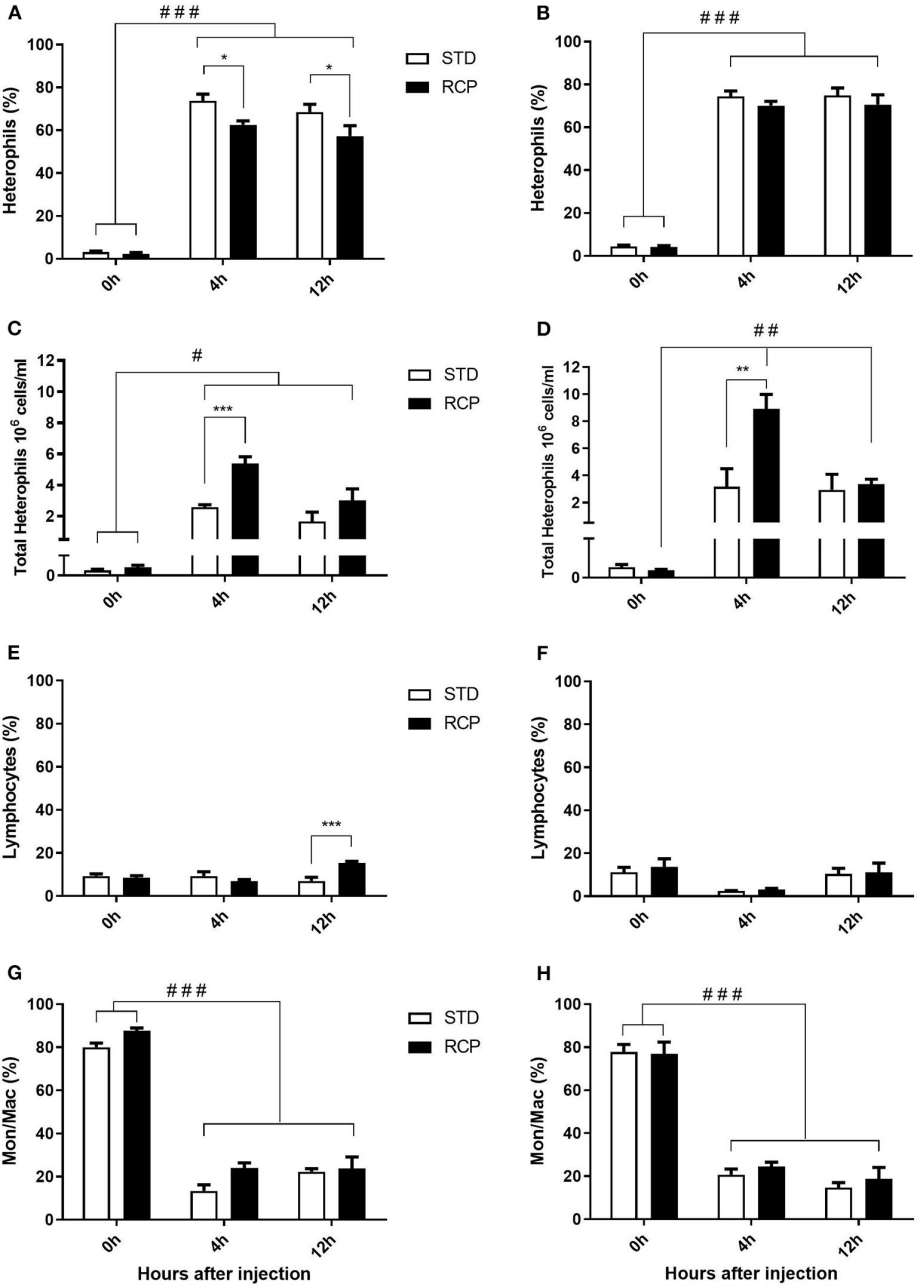
Impact of reduced crude protein diet on heterophil, lymphocyte, and monocyte/macrophage intra-abdominal leukocyte composition following an *in vivo* zymosan challenge. Changes to intra-abdominal heterophil percentages [d 14 **(A)**; d 35 **(B)**] and absolute numbers [d 14 **(C)**; d 35 **(D)**]. Parallel changes to the percentage of lymphocytes d 14 **(E)** and d 35 **(F)**, and monocyte/macrophages d 14 **(G)** and d 35 **(H)**. Heterophils are the dominant cells recruited after *in vivo* challenge and most affected by RCP diet (*N* = 5 for time 0 and 12, and *N* = 6 for time 4 h). *P < 0.05, **P < 0.01, and ***P < 0.001 between dietary treatments and ^#^*P* < 0.05, ^*##*^*P* < 0.01, and ^*###*^*P* < 0.001 among different time points. RCP, Reduced Crude Protein; STD, Standard diet.

**Figure 4:**
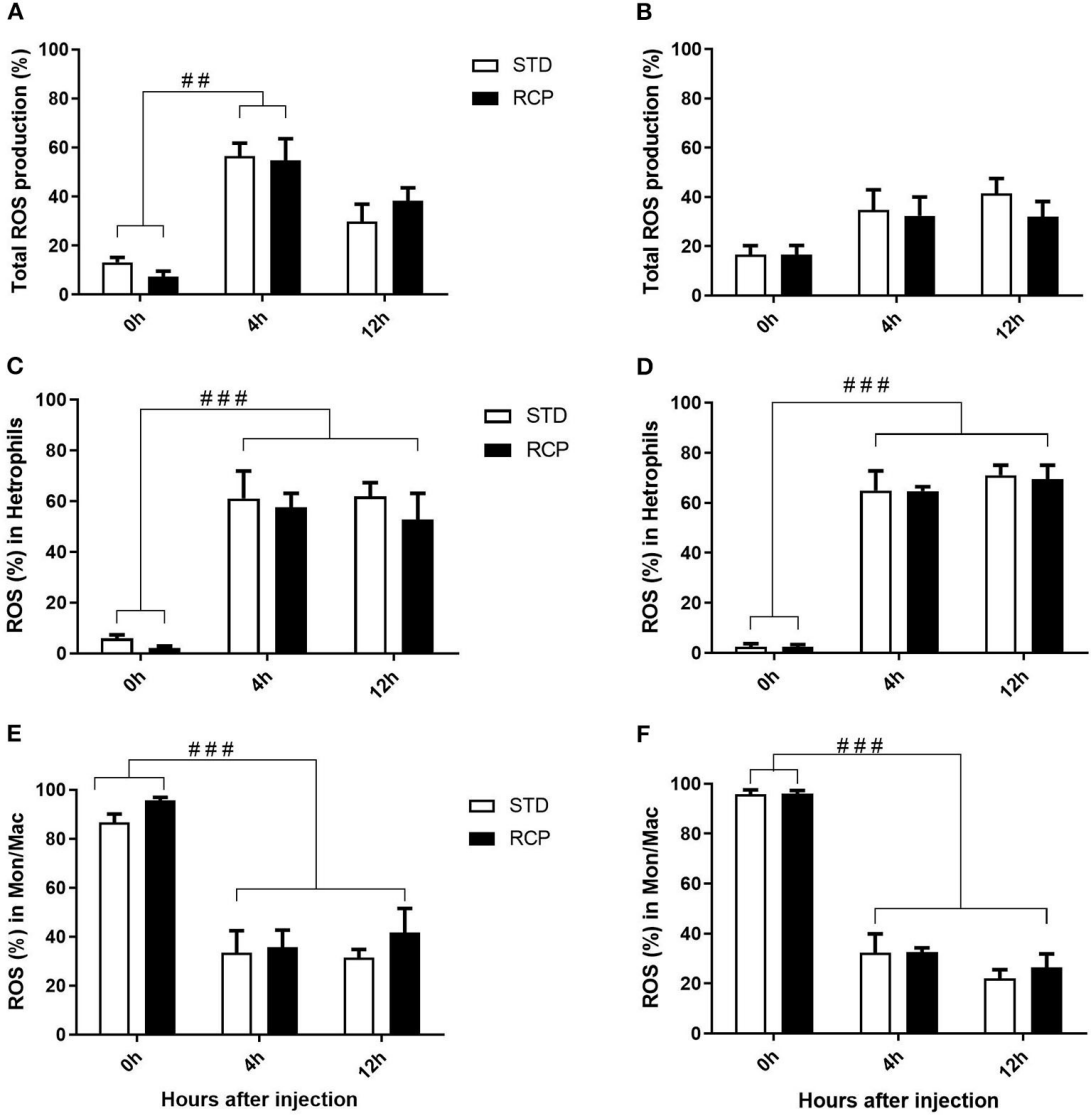
Impact of reduced crude protein diet on ROS production in abdominal leukocytes following an *in vivo* zymosan challenge. Total percentage of ROS producing cells are shown following *in vivo* challenges on day 14 **(A)** or 35 **(B)**. Higher resolution evaluation shows ROS production among distinct phagocyte subsets [heterophil d14 **(C)** and d35 **(D)**; monocyte/ macrophages d14 **(E)** and d35 **(F)**] (*n* = 5 for time 0 and 12, and *n* = 6 for time 4 **H**). ##*P* < 0.01 and ###*P* < 0.001 among different time points. RCP, Reduced Crude Protein; STD, Standard diet.

*In vivo* immune challenge using zymosan as a fungal mimic resulted in a significant increase in leukocyte recruitment to the abdominal cavity among birds supplemented with RCP in the starter phase (*P* < 0.01 compared to STD treatment; Figures 2A,B). Also, significant interaction between time and treatment was observed (*P* < 0.05), evident at both d 14 and d 35 (Figures 2A–D). We have previously shown that heterophils are the dominant leukocyte recruited into the abdominal cavity following zymosan challenge in chickens (18). These cells fill a role as the first responders against bacterial, fungal, and viral infections (30). Heterophils transmigrate into the site of inflammation in large numbers to neutralize pathogens and promote the clearance of cellular debris by phagocytosis (31–34). As expected, we found that leukocyte infiltration was dominated by heterophils (Figures 3A,B; *P* < 0.01). The higher leukocyte migration in the RCP group at d 14 correlated with higher basal levels of naïve mature neutrophils within the bone marrow storage pool (0 h; Figures 2C, **6C**), as well as significantly higher rates of leukocyte egression (Figure 2C). The higher leukocyte migration in the RCP group at d 35 correlated with higher basal numbers of total leukocytes in blood at d 35 (Figure 2F). The percentage of ROS producing leukocytes was not affected by treatments at 0, 4, and 12 h after challenge by zymosan (Figures 4A,B).

### ROS Production in Heterophils and Monocyte/Macrophage Subsets

Research is limited regarding the effects of CP level on ROS production in chicken leukocytes. A previous study reported no differences in ROS production in 4-week-old broiler's blood polymorphonuclear leukocytes fed 16 or 18% CP (35) and no differences in NO production in abdominal macrophage in broiler breeders at the age of 65 weeks fed 16 or 18% CP diet (36). Little emphasis was placed on this given that the long-standing dogma was that avian heterophils relied primarily on oxygen-independent mechanisms for antimicrobial activity (33, 34). However, our group recently showed that this is not the case, instead providing evidence for significant ROS production by chicken heterophils using a self-resolving peritonitis induced by zymosan injection (18). Consistent with these results, our current study found that heterophils dominated the leukocyte recruitment into the abdominal cavity following an equivalent zymosan challenge (Figure 3). Further, heterophils were the primary contributors to ROS production during the acute inflammatory process induced by zymosan injection (Figure 4). Although ROS production remained consistent on a per cell basis (Figure 4), the greater numbers of heterophils seen infiltrating into the immune challenge site of RCP supplemented birds point to an overall greater ROS production capacity by these cells in broiler chickens.

### Bone Marrow and Peripheral Blood Leukocyte Populations

**Figure 5:**
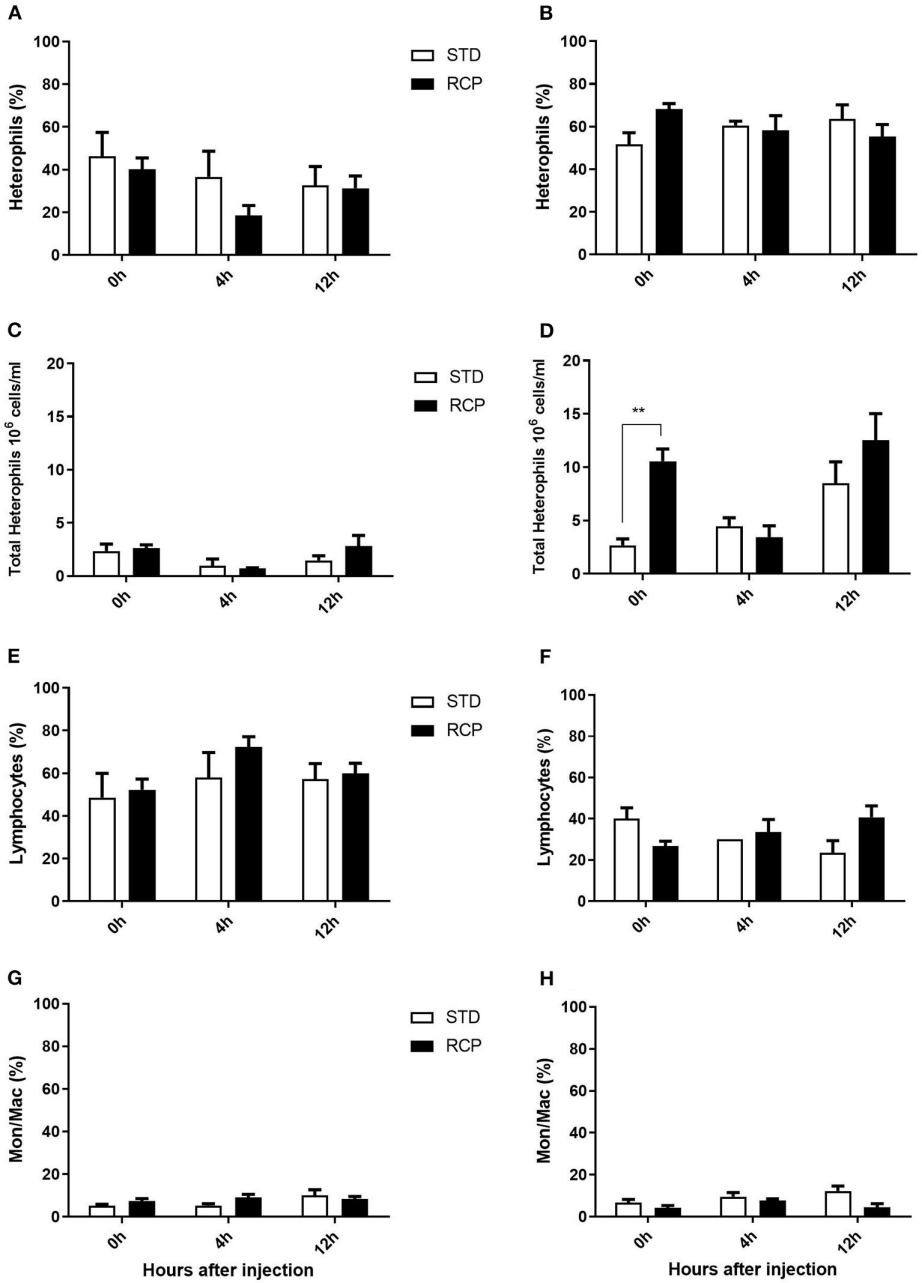
Impact of reduced crude protein diet on heterophil, lymphocyte, and monocyte/macrophage on peripheral blood leukocyte composition following an *in vivo* zymosan challenge. Changes to blood heterophil percentages [d 14 **(A)**; d 35 **(B)**] and absolute numbers [d 14**(C)**; d 35 **(D)**]. Parallel changes to the percentage of lymphocytes d 14 **(E)** and d 35 **(F)**, and monocyte/macrophages d14 **(G)** and d 35 **(H)**. **P < 0.01 between different treatments. RCP, Reduced Crude Protein; STD, Standard diet; *n* = 5.

**Figure 6:**
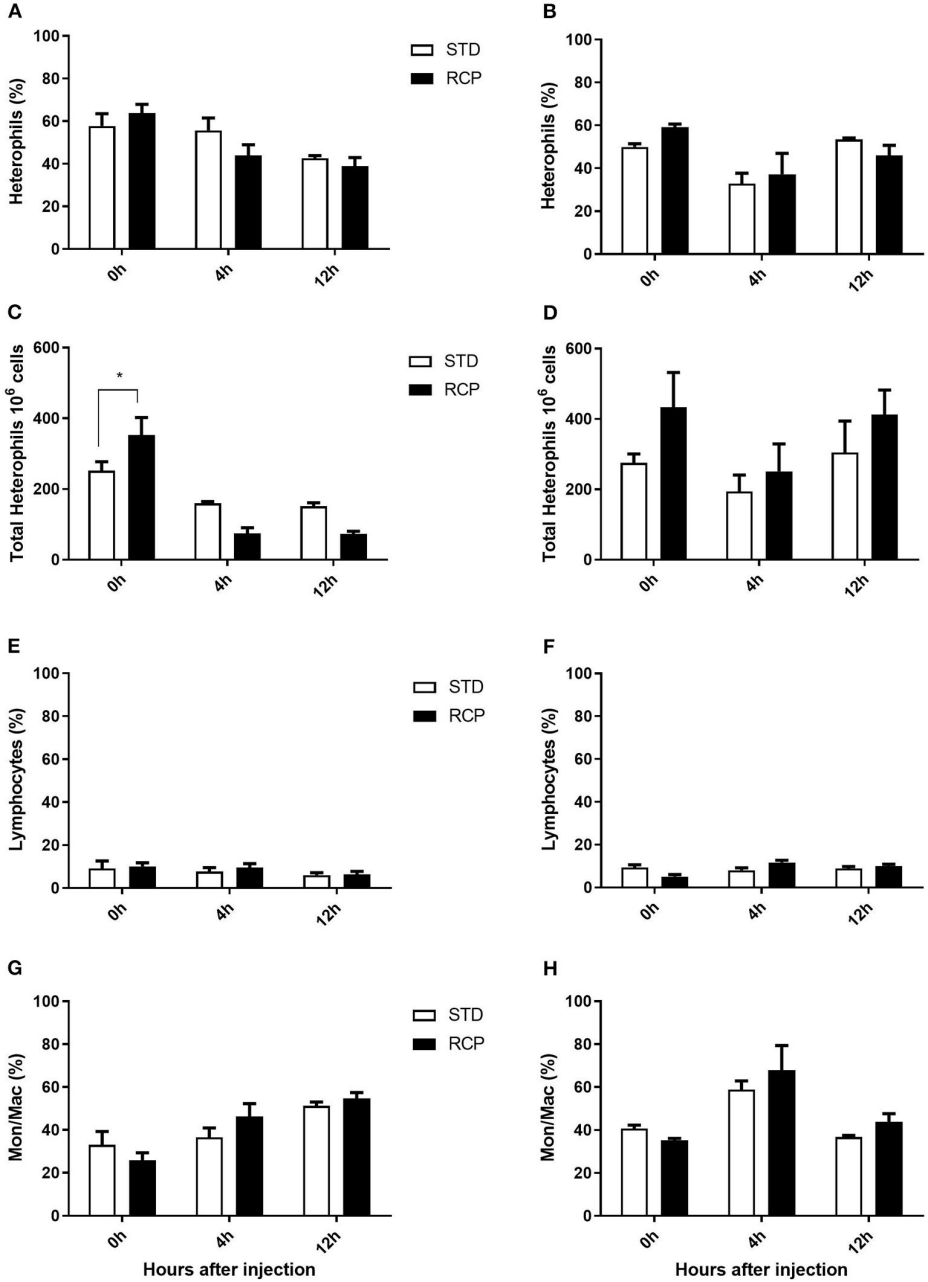
Impact of reduced crude protein diet on heterophil, lymphocyte, and monocyte/macrophage on bone marrow leukocyte composition following an *in vivo* zymosan challenge. Bone marrow leukocyte subpopulation after 0, 4, and 12 h post intra-abdominal administration of zymosan (2.5 mg). Changes to bone marrow heterophil percentages [d 14 **(A)**; d 35 **(B)**] and absolute numbers [d 14 **(C)**; d 35 **(D)**]. Parallel changes to the percentage of lymphocytes d 14 **(E)** and d 35 **(F)**, and monocyte/macrophages d14 **(G)** and d 35 **(H)**. *P < 0.05 between different treatments. RCP, Reduced Crude Protein; STD, Standard diet; *n* = 5.

The bone marrow offers a hematopoietic compartment where immune cells initially develop from early progenitors and stores of mature naïve leukocytes are maintained, prior to their release into blood circulation (5, 6, 18). Thus, these represent relevant organ sites to evaluate the distribution and migration of white blood cells upon an immune challenge. Impact of reduced crude protein diet on peripheral blood and bone marrow leukocyte composition following an *in vivo* zymosan challenge is shown in Figures 5, 6, respectively. Chickens fed RCP and STD diets for 14 days showed equivalent basal numbers of leukocytes (i.e., pre-immune challenge) in the abdominal cavity, bone marrow, and peripheral blood (Figures 2A,C,E). Basal numbers remained consistent in the abdominal cavity and bone marrow after 35 days of growth (Figures 2B,D). However, significantly higher basal numbers of leukocytes were identified in the blood on d 35 (*P* < 0.01; Figure 2F). Consistent with these results, higher resolution examination of distinct heterophil, lymphocyte, and monocyte/macrophage subsets showed significantly higher numbers of heterophils in the blood after 35 days of growth (Figure 5). Upon zymosan challenge, the RCP group demonstrated lower total leukocyte numbers but no change in the proportion of individual subsets in bone marrow on d 14 (Figures 2C, 6 respectively) after 4 h of intra-abdominal challenge (*P* < 0.01). This was likely associated with higher leukocyte egression from this hematopoietic compartment in route to the abdominal challenge site (Figure 2). However, this significant change in bone marrow total leukocyte was not observed on d 35 (Figure 2D). Interestingly, we detected a higher capacity for total leukocyte recruitment into the abdominal cavity at d 35 compared to d 14 (Figures 2B vs. 2A), which further correlated with higher basal levels of leukocytes in the blood for the same time periods (Figures 2F vs. 2E). One possibility is that the blood may play a greater role in the maintenance of leukocytes that can be recruited to a site of infection at later stages of bird development. Thus, higher availability of blood leukocytes and subsequent infiltration to the challenge site may reflect a potential for enhanced protection among RCP birds.

Indirect hormonal influences of dietary protein may be a contributing factor for increased leukocyte levels in the RCP group. Changes in the endocrine system and metabolic activities, which is directly influenced by diet, have been shown to affect immune status (37). Many components in the endocrine system, including growth hormone, thyroid hormones, and corticosterone levels, can be regulated under protein or energy restriction condition (19, 37, 38). Additionally, it has been demonstrated that heterophil release from bone marrow to the circulatory blood are enhanced under corticosterone stimulation (39). Therefore, it is reasonable to suggest that the increase in leukocyte and heterophils in bone marrow, blood, and subsequently higher leukocyte infiltration into the abdominal cavity may be the consequences of changes in the endocrine system in response to the lower crude protein level, although future study is needed to clarify the link between CP level, endocrine system, and immune modulation.

### Proinflammatory Cytokines Gene Expression

**Figure 7:**
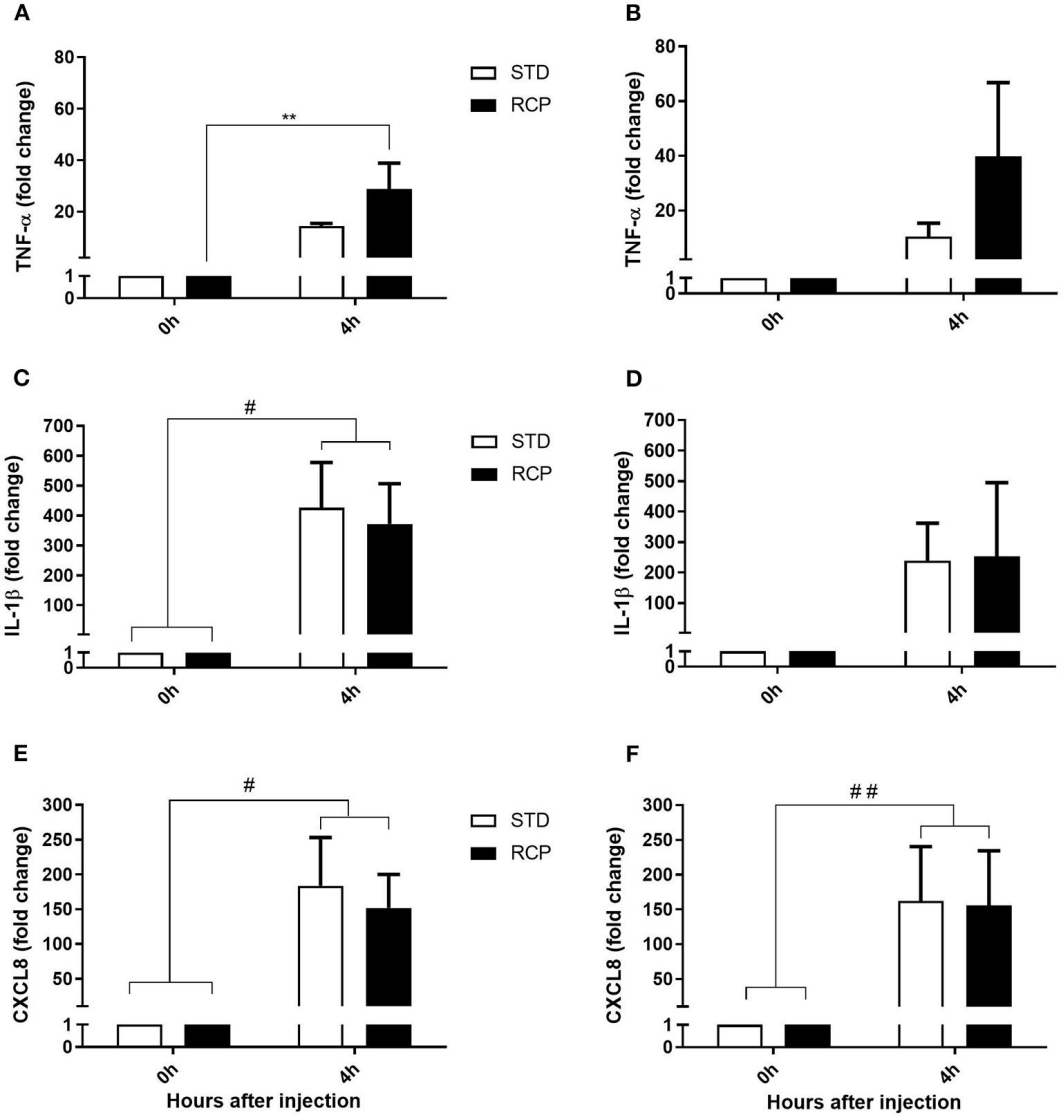
Impact of reduced crude protein diet on gene expression of pro-inflammatory markers following an *in vivo* zymosan challenge. Fold change gene expression in TNF-α on d 14 **(A)** and d 35 **(B)**, IL-1β on d14 **(C)**, and d 35 **(D)**, CXCL8 on d 14 **(E)**, and d 35 **(F)** in abdominal leukocytes after 0 and 4 h intra-abdominal challenge with zymosan. *P < 0.05 between different treatments and #*P* < 0.05 and ##*P* < 0.01 among different time points. RCP, Reduced Crude Protein; STD, Standard diet; *n* = 6.

The early phase of acute inflammation is manifested by a multifaceted functionality that involves the release of lipid mediators such as prostaglandin E2, leukotriene C4, thromboxane A2, chemokines such as CXCL8, and pro-inflammatory cytokines such as TNF- α, IL-1 β, IL-6, IL-17, which ultimately contribute to the recruitment of leukocytes to the site of inflammation (17, 18). TNF-α and IL-1β represent early cytokine activators of acute inflammation, while CXCL8 is among the earliest chemokines that contribute to leukocyte recruitment. Thus, these three genes were used as molecular markers of the chicken acute inflammatory response. No differences were detected in the basal expression of these three genes (Figure 7) between STD and RCP group (*P* > 0.05). In contrast, higher levels of TNF-α expression (Figure 7A) were detected in the RCP group after intra-abdominal challenge with zymosan on day 14 (*P* < 0.01). Enhanced levels of TNF-α expression are consistent with the higher levels of leukocyte infiltration described above in RCP group after intra-abdominal challenge.

## Conclusions

In summary, moderate reductions in dietary CP in the starter phase of broilers did not significantly affect growth performance or relative organ weights in either the starter phase or beyond until 35 days of age. This was to be expected as diets were formulated to meet all standard ileal digestible essential amino acid requirements in optimal ratios. However, compared to the STD treatment, RCP in the starter phase significantly increased leukocyte recruitment to the abdominal cavity in response to an *in vivo* immune challenge using zymosan as a fungal mimic. This was observed during the starter phase up to day 14 of age, after which all birds were fed a common standard crude protein grower diet. Additionally, the total leukocyte levels in the blood at day 35 in unchallenged treatments were higher in the RCP compared to STD groups. These changes persisting into the grower period may point to epigenetic changes affecting immune development in response to the changes in nutrition occurring directly post-hatch during the developmental starter phase. It remains to be investigated whether the observed immune developmental changes are a direct consequence of the dietary protein levels, or reflect differences in the availability of free vs. protein bound amino acids between the diets, and/or due to the differences in starch:lipid ratios of the diets as a result of the changes to raw ingredient inclusion to reduce CP. While no difference in the inflammatory mediators or ROS production could be observed, it is hypothesized that the time points for observing these changes potentially occurred earlier than the first samplings. Future research could involve additional earlier time points, as well as additional inflammatory markers to further understand the drivers and subsequent changes in immune activity, in response to the dietary CP levels in the starter phase. The industry would also benefit from further research, to investigate how the immune changes occurring from RCP in the starter phase may benefit vaccination strategies, disease resistance to different kinds of field relevant pathogens, or growth performance recovery post-challenge. In conclusion, this study provides exciting new evidence to support the potential benefits of starter phase RCP diets for the development and response of the immune system in broilers.

## Data Availability Statement

The datasets generated for this study are available on request to the corresponding author.

## Ethics Statement

All animals were maintained according to the guidelines specified by the Canadian Council on Animal Care, and protocols were approved by the University of Alberta Animal Care and Use Committee.

## Author Contributions

MK, JW, RW, VN, and DB conceived and designed the study. MK and WH performed the experiments. MK analyzed and interpreted the data, prepared the figures, and drafted the manuscript. All authors participated in reviewing the manuscript and approved the final version of the manuscript. All authors contributed to the article and approved the submitted version.

## Conflict of Interest

RW and VN were employed by Evonik Nutrition & Care GmbH. This work was supported by the Evonik Nutrition & Care GmbH grant RES0037933. The funder contributed to study design and manuscript review. The funder was not involved in collection, analysis, or interpretation of data. The remaining authors declare that the research was conducted in the absence of any commercial or financial relationships that could be construed as a potential conflict of interest.

## Abbreviations

AMEn: Apparent metabolizable energy nitrogen-corrected
CP: Crude protein
FCR: Feed conversion ratio
Mon/Mac: Monocyte/macrophages
RCP: Reduced crude protein diet
ROS: Reactive oxygen species
SID: Standard ileal digestible
STD: Standard diet.
